# Supplementary material for: BPTF regulates androgen receptor activity by enhancing chromatin accessibility and stabilizing the AR-FOXA1 interaction
Source: Nat Commun. 2025 Dec 11;17:670. doi: 10.1038/s41467-025-67329-9 (PMC12820202; doi:10.1038/s41467-025-67329-9)
Supplement: Supplementary file 1 — Supplementary Information [file 41467_2025_67329_MOESM1_ESM.pdf]

BPTF regulates androgen receptor activity by enhancing chromatin accessibility and stabilizing the AR-FOXA1 interaction

Hee-Young Jeon, Sudeep Khadka, Majid Pornour, Hyunju Ryu, Hegang Chen, Arif Hussain, Hung-Ming Lam, Eva Corey, Htoo Zarni Oo, Martin Gleave, Xiaofang Che, Christopher Barbieri, Jianfei Qi

Supplementary Figures

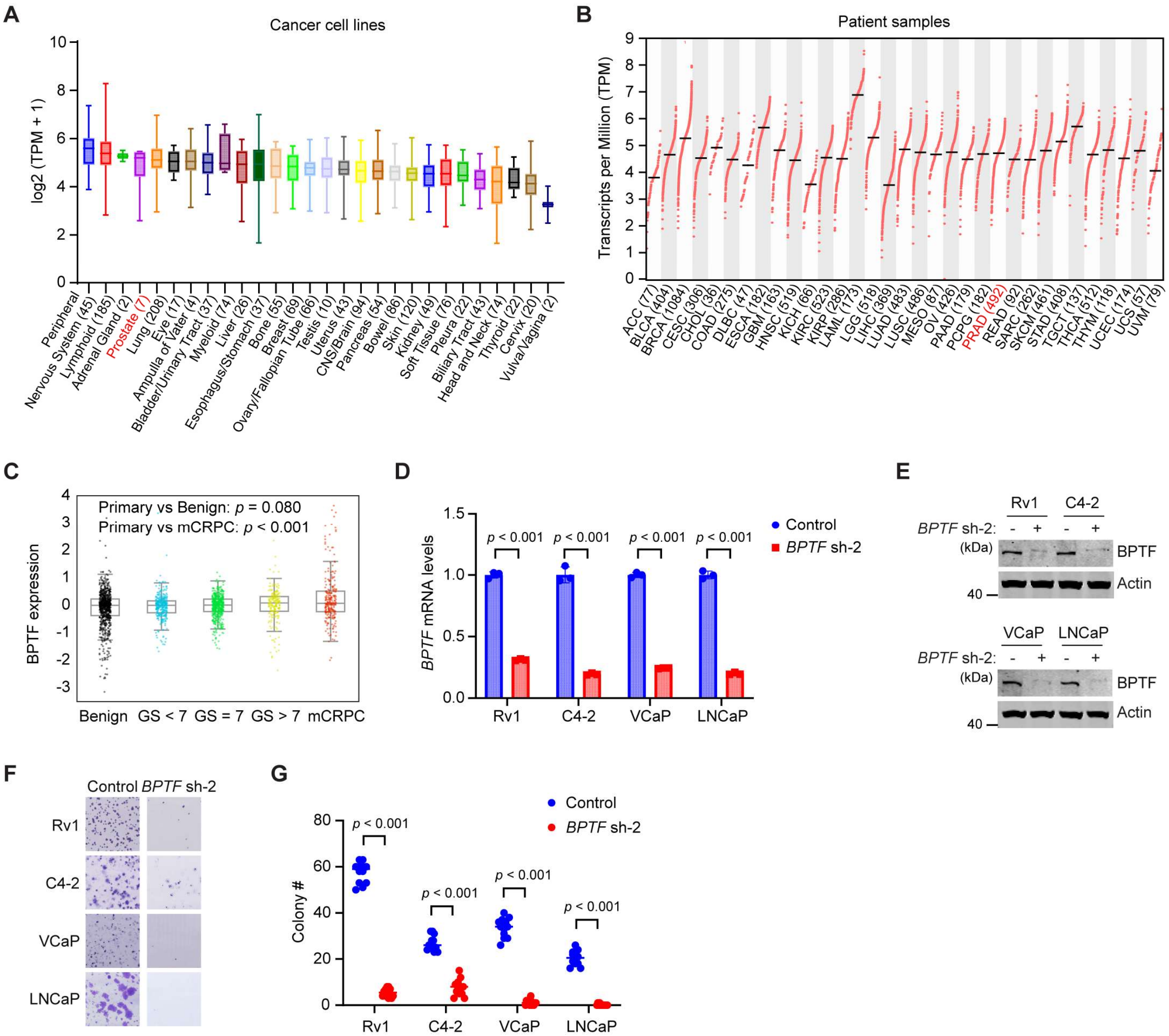

Fig. S1. *BPTF* expression in various cancer types and inhibition of PCa cell growth by a second *BPTF* shRNA. **A** *BPTF* mRNA expression across various cancer cell lines. Data were downloaded from DepMap Portal and sorted by the median expression value for each cancer types. The PCa cell line is highlighted in red. **B** *BPTF* mRNA expression in tissues of different cancer types from GEPIA2. PRAD (prostate adenocarcinoma) is highlighted in red. **C.** *BPTF* mRNA expression in benign prostate, primary PCa (Gleason score <7, =7, or >7) and CRPC samples from PCTA (Prostate Cancer Transcriptome Atlas). **D, E** The knockdown (KD) efficiency of a second *BPTF* shRNA (sh-2) in PCa cells is evaluated by qRT-PCR (**D**) and western blot analysis (**E**). **F, G** Colony formation assay of PCa cells following BPTF KD with BPTF shRNA (sh-2). Representative images (**F**) and quantification (**G**) are shown. Data are representative of three independent biological replicates (**D-G**). Data are presented as mean ± SD (**D**). Statistical significance was determined using a two-tailed unpaired Student's t-test (**D**, **G**). Source data are provided as a Source Data file.

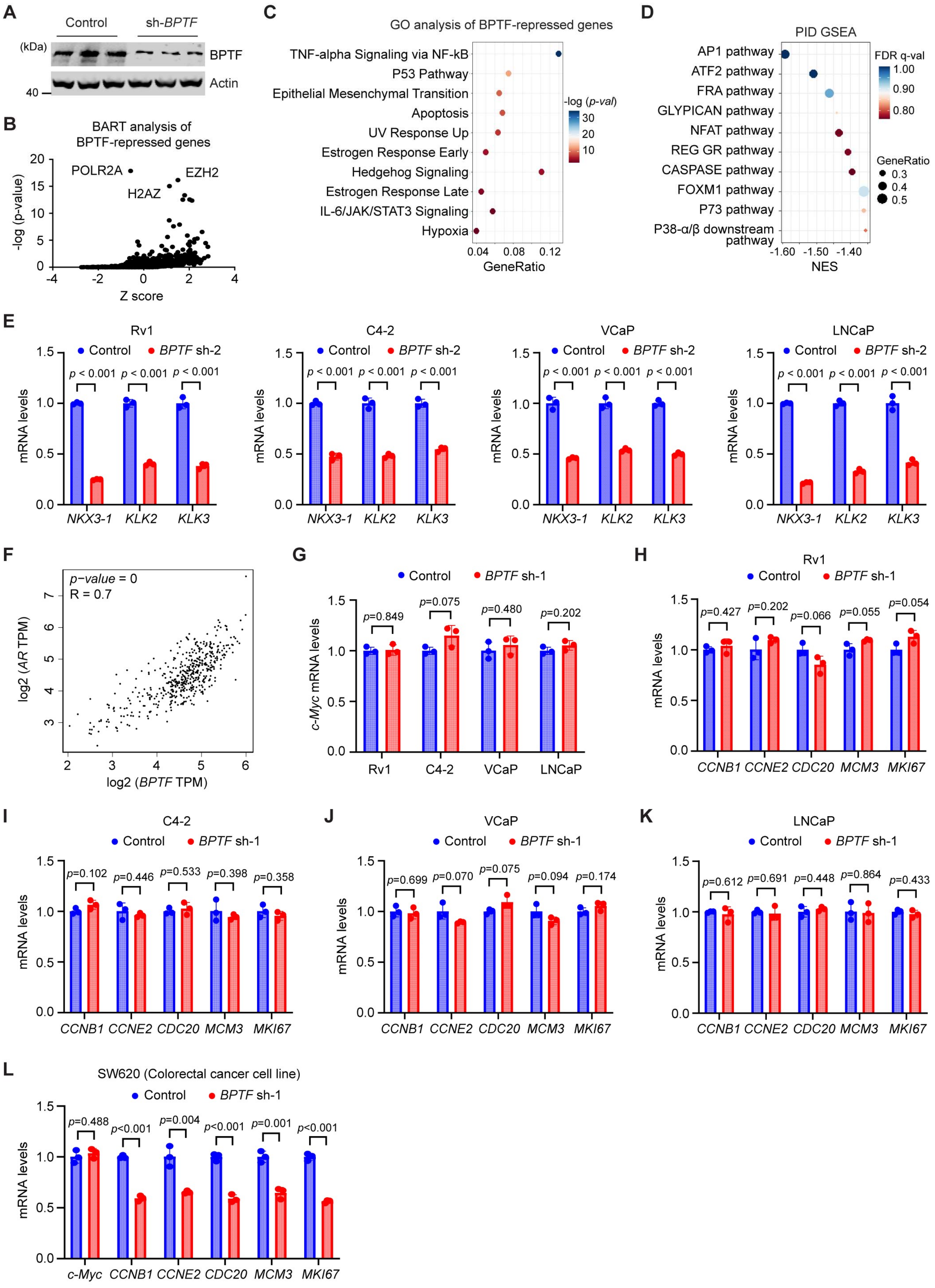
A
Control
sh-BPTF
(kDa)
BPTF
Actin
40
B
BART analysis of
BPTF-repressed genes
-log (p-value)
POLR2A
H2AZ
EZH2
Z score
C
GO analysis of BPTF-repressed genes
TNF-alpha Signaling via NF-kB
P53 Pathway
Epithelial Mesenchymal Transition
Apoptosis
UV Response Up
Estrogen Response Early
Hedgehog Signaling
Estrogen Response Late
IL-6/JAK/STAT3 Signaling
Hypoxia
-log (p-val)
GeneRatio
D
PID GSEA
AP1 pathway
ATF2 pathway
FRA pathway
GLYPICAN pathway
NFAT pathway
REG GR pathway
CASPASE pathway
FOXM1 pathway
P73 pathway
P38-α/β downstream pathway
FDR q-val
GeneRatio
NES
E
Rv1
C4-2
VCaP
LNCaP
Control
BPTF sh-2
p < 0.001
mRNA levels
NKX3-1
KLK2
KLK3
F
p−value = 0
R = 0.7
log2 (AR TPM)
log2 (BPTF TPM)
G
Control
BPTF sh-1
c-Myc mRNA levels
p=0.849
p=0.075
p=0.480
p=0.202
Rv1
C4-2
VCaP
LNCaP
H
Rv1
p=0.427
p=0.202
p=0.066
p=0.055
p=0.054
CCNB1
CCNE2
CDC20
MCM3
MKI67
I
C4-2
p=0.102
p=0.446
p=0.533
p=0.398
p=0.358
J
VCaP
p=0.699
p=0.070
p=0.075
p=0.094
p=0.174
K
LNCaP
p=0.612
p=0.691
p=0.448
p=0.864
p=0.433
L
SW620 (Colorectal cancer cell line)
p=0.488
p<0.001
p=0.004
p<0.001
p=0.001
p<0.001
c-Myc

Fig. S2. BPTF upregulates the expression of AR target genes but has little effect on c-Myc target gene expression in PCa cells. **A** Reduced BPTF protein levels in BPTF-KD Rv1 cells used for RNA-seq analysis. **B** BART analysis of BPTF-repressed genes (log_2 [fold change] > 1, *Padj* < 0.05, following BPTF KD in Rv1 cells). **C** GO analysis of BPTF-repressed genes (log_2 [fold change] > 0.8, *Padj* < 0.05, following BPTF KD in Rv1 cells), based on the MSigDB hallmark gene set. **D** GSEA of DEGs after BPTF KD in Rv1 cells, based on the Pathway Interaction Database (PID). Only pathways enriched in BPTF-KD cells are shown. **E** Reduced mRNA levels of representative AR target genes following BPTF KD (sh-2) in PCa cells. **F** Correlation analysis of *BPTF* and *AR* mRNA expression levels in TCGA PCa dataset. **G** qRT-PCR analysis showing *c-Myc* mRNA levels following BPTF KD in PCa cells. **H-K** qRT-PCR analysis showing mRNA levels of representative c-Myc target genes following BPTF KD in PCa cells. **L** qRT-PCR analysis showing mRNA levels of *c-Myc* and representative c-Myc target genes in SW620 colorectal cancer cells following BPTF KD. Data are representative of three independent biological replicates and are presented as mean ± SD (**E**, **G-L**). Statistical significance was determined using a two-tailed unpaired Student's t-test (**E, G-L**). Source data are provided as a Source Data file.

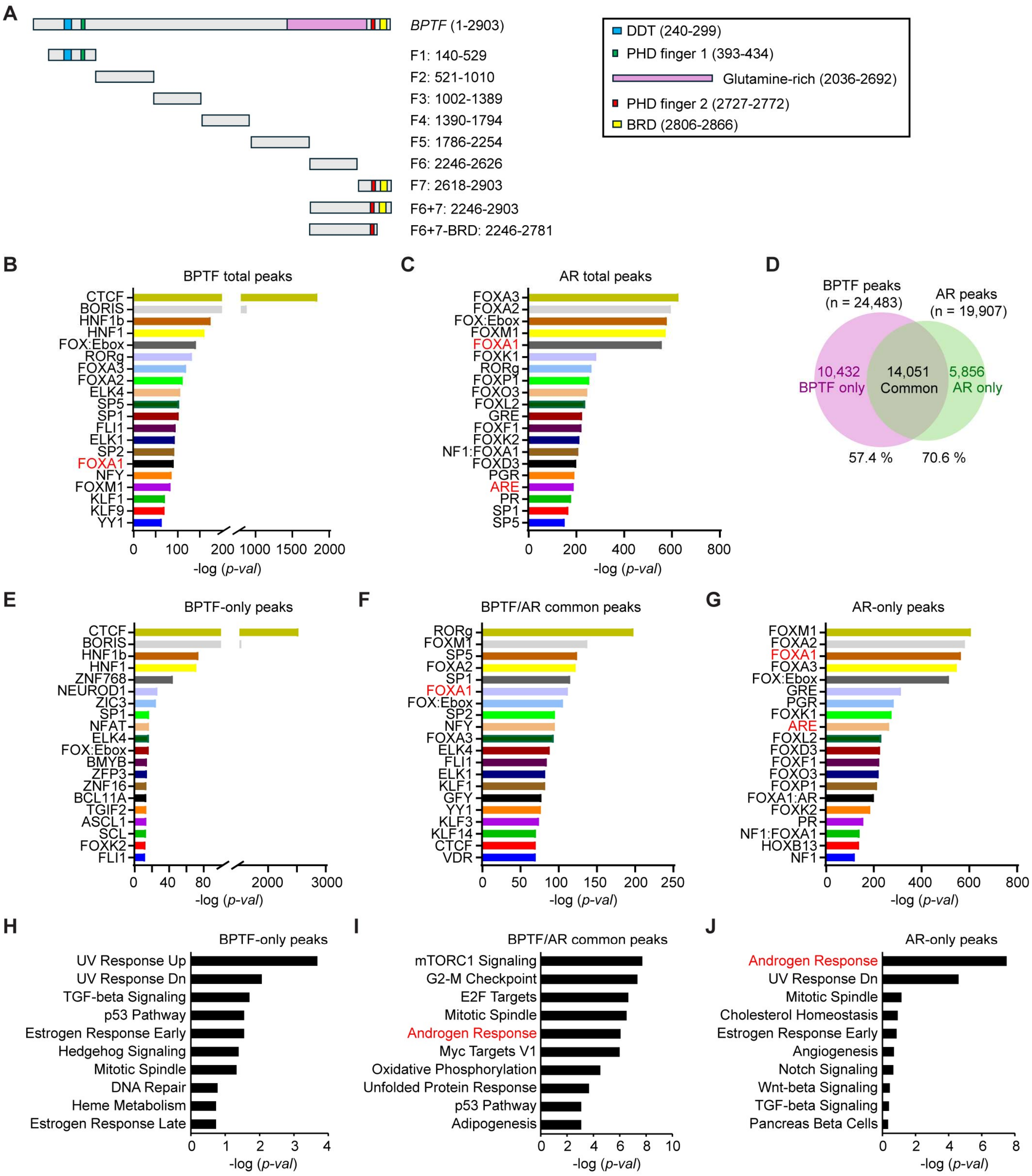

Fig. S3. Transcription factor motifs and pathways enriched in different peak categories (BPTF-only, BPTF/AR common, AR-only). **A** Schematic representation of BPTF structure, domains, and fragments. **B**, **C** HOMER motif analysis of 24,483 total BPTF peaks (**B**) and 19,907 total AR peaks (**C**), showing the top 20 enriched transcription factor motifs. **D** Venn diagram showing the peak categories: BPTF-only, BPTF/AR common and AR-only peaks. **E-G** Motif analysis of BPTF-only (**E**), BPTF/AR common (**F**), and AR-only (**G**) peaks, showing the top 20 enriched transcription factor motifs in each category. **H-J** GO analysis of genes associated with BPTF-only (**H**), BPTF/AR common (**I**), and AR-only (**J**) peaks.

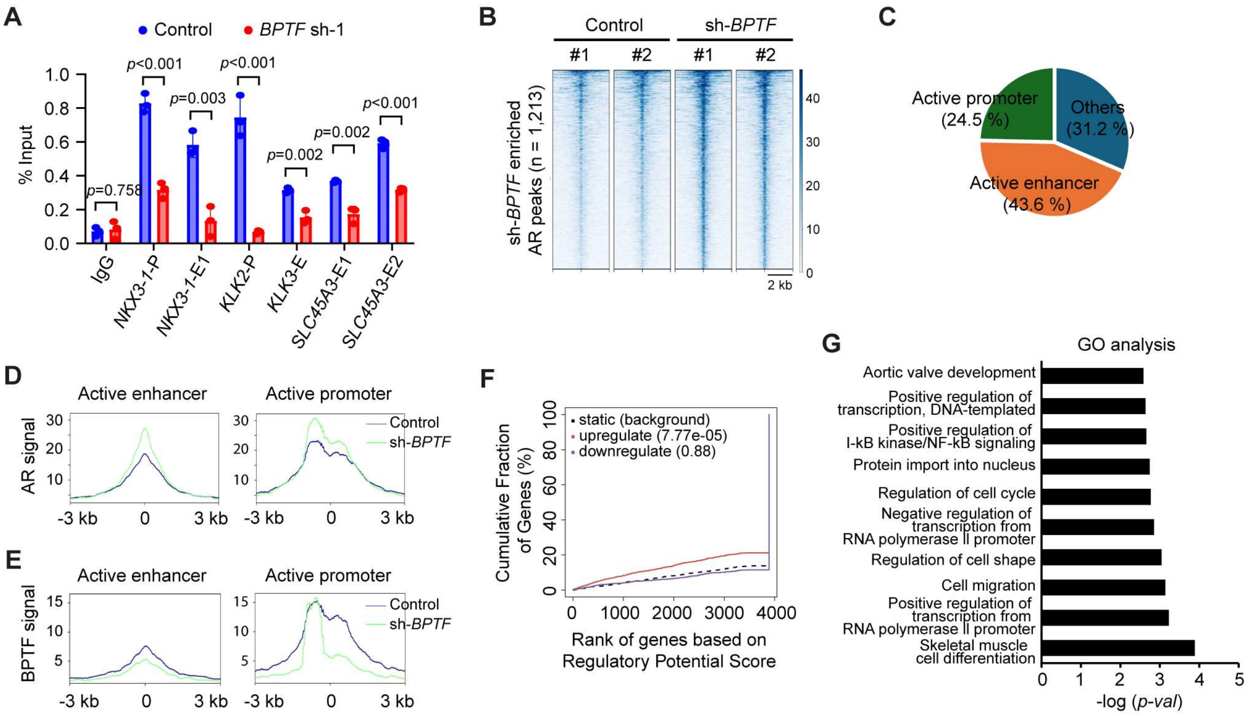

Fig. S4. Analysis of BPTF-repressed AR peaks. **A** ChIP-qPCR analysis of AR at the promoter or enhancer regions of representative AR target genes after BPTF KD in VCaP cells. **B** Heatmap of BPTF-repressed AR peaks, defined as the 1,213 AR peaks with increased signal after BPTF KD in Rv1 cells. **C** Genomic distribution of the BPTF-repressed AR peaks (n=1,213) at active enhancer and promoter regions. **D, E** Peak profile plots showing AR (**D**) and BPTF (**E**) signal intensities at BPTF-repressed AR peaks located in active enhancer or promoter regions. **F** BETA analysis showing the correlation between BPTF-repressed AR peaks and BPTF-repressed genes (i.e., transcripts upregulated in BPTF-KD RNA-seq). **G** GO analysis of direct BPTF-repressed AR target genes identified in (**F**). Data are representative of three independent biological replicates, presented as mean ± SD, and statistical significance was determined using a two-tailed unpaired Student's t-test (**A**). Source data are provided as a Source Data file.

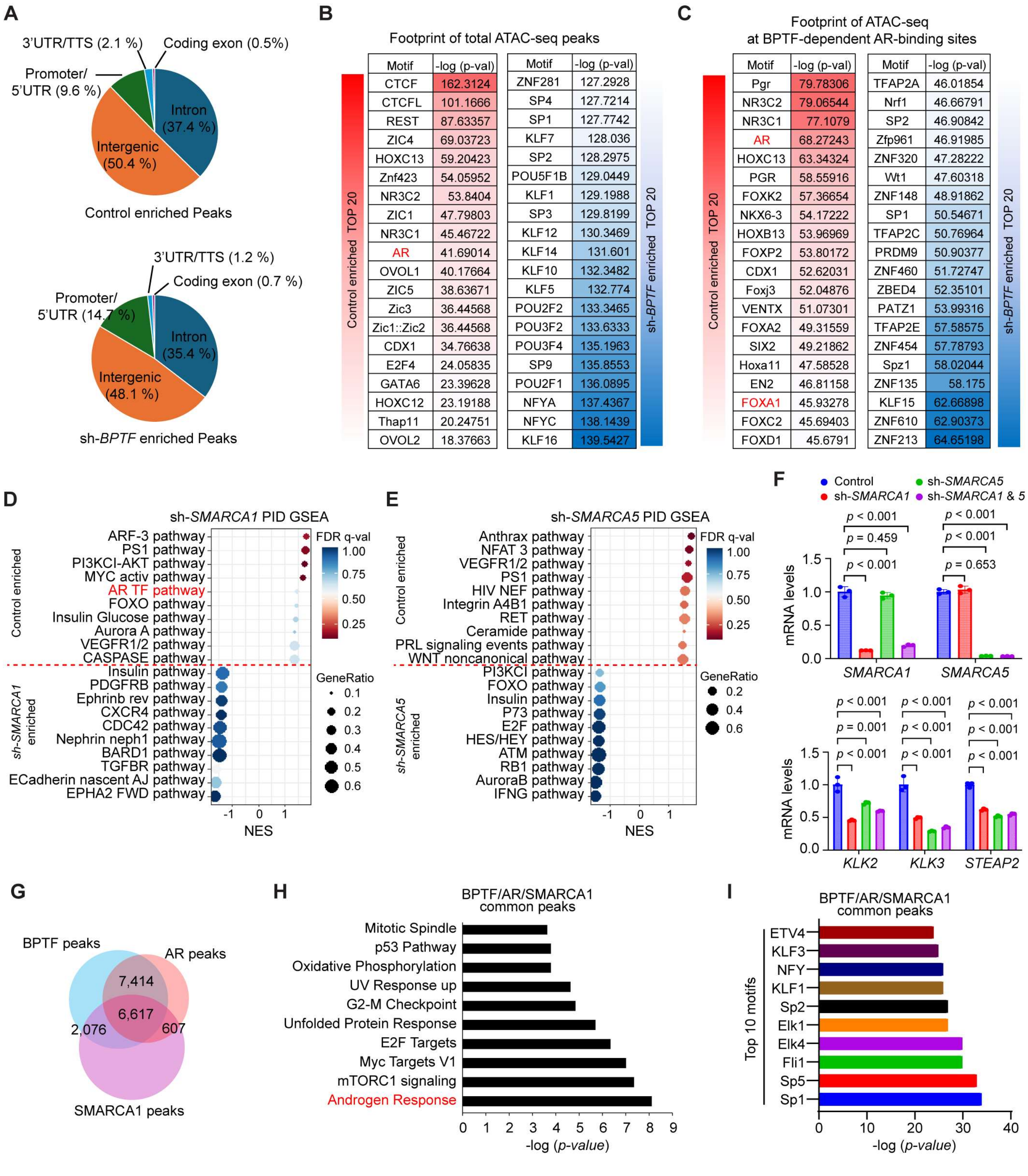

Fig. S5. BPTF enhances chromatin accessibility to facilitate AR binding**. A** Genomic distribution of differential ATAC-seq peaks in Rv1 cells following BPTF KD, including 6,944 control-enriched peaks (top) and 3,716 BPTF KD-enriched peaks (bottom). **B, C** Comparative footprint analysis of total ATAC-seq peaks (**B**) or those at BPTF-dependent AR-binding sites (**C**), highlighting the top 20 enriched transcription factor motifs in control (left) and BPTF-KD (right) Rv1 cells. **D** GSEA of DEGs using PID gene sets, showing the top 10 enriched pathways in control (top) or SMARCA1-KD (bottom) Rv1 cells. **E** GSEA of DEGs using PID gene sets, showing the top 10 enriched pathways in control (top) or SMARCA5-KD (bottom) Rv1 cells. **F** mRNA levels of

SMARCA1, *SMARCA5*, and representative AR target genes after individual or combined KD of SMARCA1 and SMARCA5 in Rv1 cells. **G** Venn diagram showing the overlap of CUT&RUN ChIP-seq peaks for BPTF, AR, and SMARCA1. **H** GO analysis of genes associated with AR/BPTF/SMARCA1 common peaks. **I** Motif analysis of AR/BPTF/SMARCA1 common peaks. Data are representative of three independent biological replicates, presented as mean $\pm$ SD, and statistical significance was determined using one-way ANOVA (**F**). Source data are provided as a Source Data file.

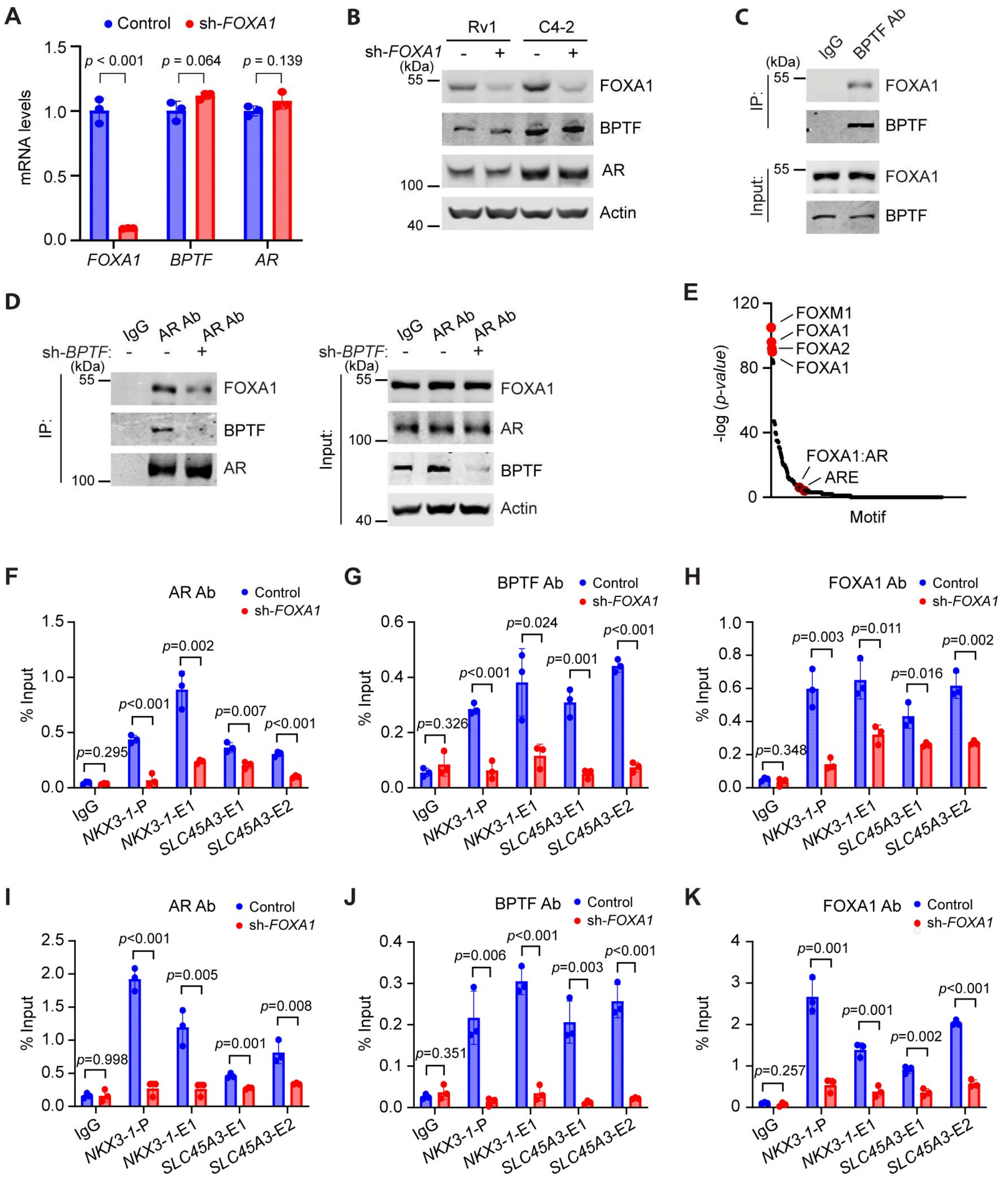

Fig. S6. BPTF interacts with FOXA1 and stabilizes AR-FOXA1 interaction on chromatin. **A** qRT-PCR analysis showing mRNA levels of *FOXA1*, *BPTF* and *AR* in Rv1 cells following *FOXA1* KD. **B** Western blots showing protein levels of FOXA1, BPTF and AR in Rv1 and C4-2 cells following FOXA1 KD. **C** Co-IP of FOXA1 with BPTF in C4-2 cells. **D** Co-IP of FOXA1 with AR in C4-2 cells following BPTF KD. **E** Motif analysis of peaks co-occupied by BPTF, AR, and FOXA1. **F-H** ChIP-qPCR analysis showing enrichment of AR (**F**), BPTF (**G**), and FOXA1 (**H**) at promoter or enhancer regions of representative AR target genes in Rv1 cells following FOXA1 KD. **I-K** ChIP-qPCR analysis showing enrichment of AR (**I**), BPTF (**J**), and FOXA1 (**K**) at promoter or enhancer regions of representative AR target genes in VCaP cells following FOXA1 KD. Data are representative of three independent biological replicates (**A-D, F-K**). Data are

presented as mean ± SD, and statistical significance was determined using a two-tailed unpaired Student's t-test for **(A, F-K)**. Source data are provided as a Source Data file.

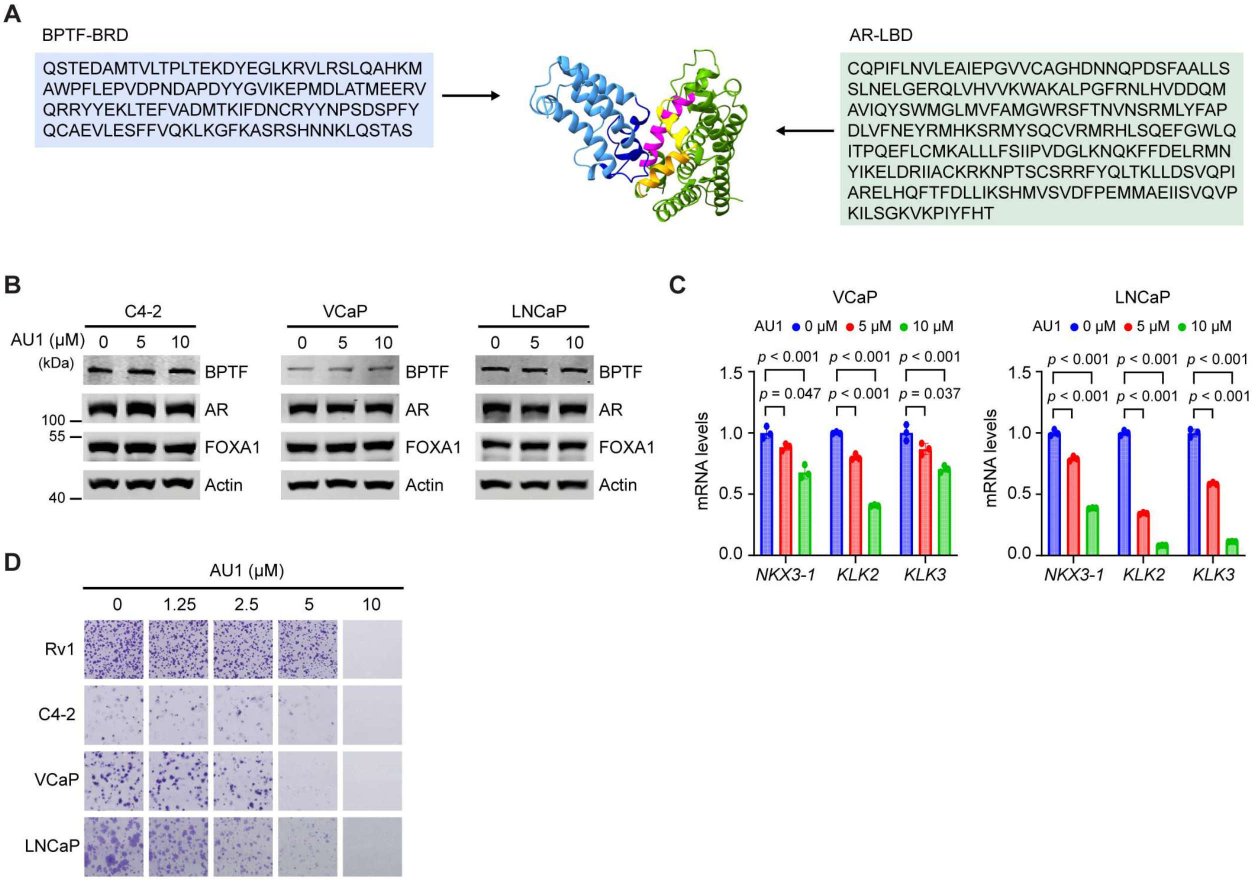

Fig. S7. Effect of AU1 on AR activity and proliferation of PCa cells. **A** Amino acid sequence of BPTF-BRD and AR-LBD used for predicting the 3D interaction model with AlphaFold2. **B** Western blot analysis showing protein levels of BPTF, AR, and FOXA1 in PCa cells after AU1 treatment for 24 hours. **C** qRT-PCR analysis showing mRNA levels of representative AR target genes after treatment of VCaP or LNCaP cells with AU1 for 24 hour. **D** Example images of colony formation of PCa cells in the presence of increasing concentrations of AU1. Data are representative of three independent biological replicates (**B-D**). Data are presented as mean ± SD, and statistical significance was determined using one-way ANOVA (**C**). Source data are provided as a Source Data file.

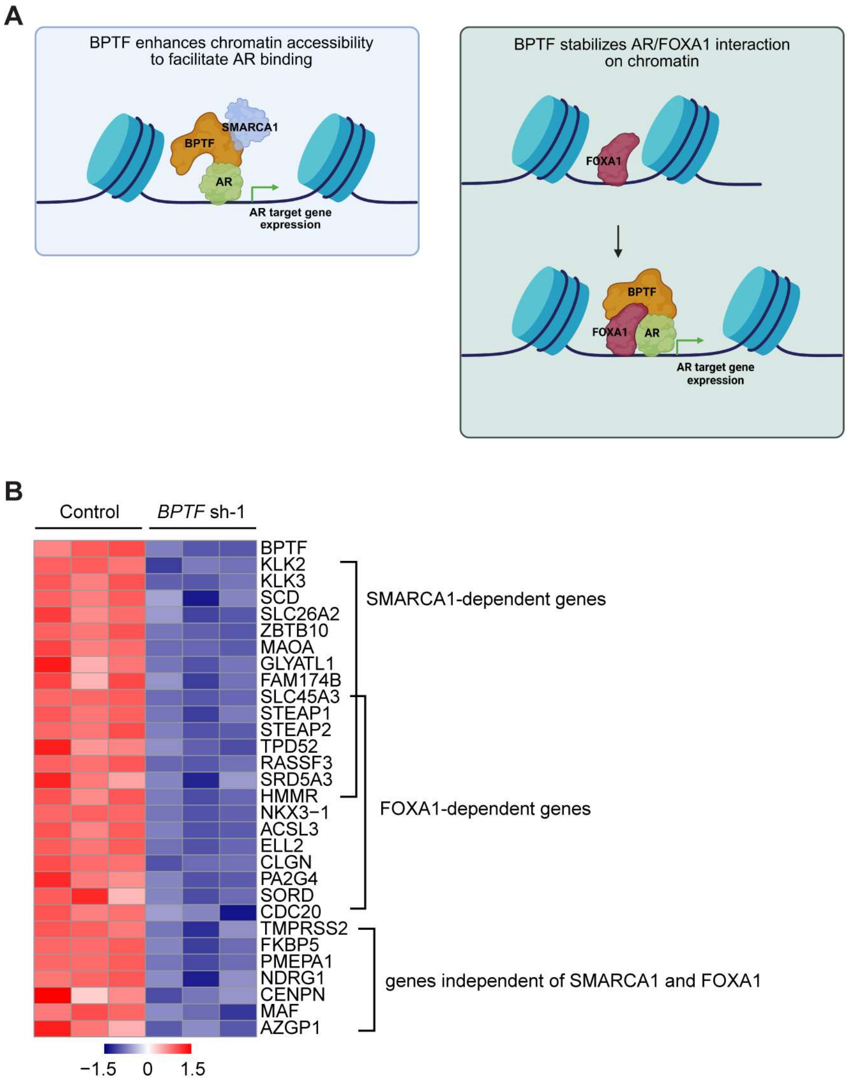

Fig. S8. A Schematic models illustrating the two mechanisms by which BPTF promotes AR activity. Created in BioRender. JEON, H. (2025) https://BioRender.com/xcyrkey. **B** Heatmap of BPTF-dependent AR hallmark target genes, highlighting genes that are SMARCA1-dependent, FOXA1-dependent, or independent of both SMARCA1 and FOXA1.
