# Supplementary material for: BPTF regulates androgen receptor activity by enhancing chromatin accessibility and stabilizing the AR-FOXA1 interaction
Source: Nat Commun. 2025 Dec 11;17:670. doi: 10.1038/s41467-025-67329-9 (PMC12820202; doi:10.1038/s41467-025-67329-9)
Supplement: Supplementary file 2 — Description of Additional Supplementary Files [file 41467_2025_67329_MOESM2_ESM.docx]

**Description of additional supplementary files**

**Name:** Supplementary Data 1

**Description:** Differentially expressed genes (DEGs) (padj <0.05) following BPTF knockdown in Rv1 cells.

**Name:** Supplementary Data 2

**Description:** List of proteins identified by AR RIME.

**Name:** Supplementary Data 3

**Description:** Antibodies and working dilutions.
