# Supplementary material for: BPTF regulates androgen receptor activity by enhancing chromatin accessibility and stabilizing the AR-FOXA1 interaction
Source: Nat Commun. 2025 Dec 11;17:670. doi: 10.1038/s41467-025-67329-9 (PMC12820202; doi:10.1038/s41467-025-67329-9)
Supplement: Supplementary file 6 — Reporting Summary [file 41467_2025_67329_MOESM6_ESM.pdf]

nature portfolio

Corresponding author(s): Jianfei Qi

Last updated by author(s): Nov 19, 2025

Reporting Summary

Nature Portfolio wishes to improve the reproducibility of the work that we publish. This form provides structure for consistency and transparency in reporting. For further information on Nature Portfolio policies, see our Editorial Policies and the Editorial Policy Checklist.

Statistics

For all statistical analyses, confirm that the following items are present in the figure legend, table legend, main text, or Methods section.

n/a	Confirmed	
☐	☒	The exact sample size (*n*) for each experimental group/condition, given as a discrete number and unit of measurement
☐	☒	A statement on whether measurements were taken from distinct samples or whether the same sample was measured repeatedly
☐	☒	The statistical test(s) used AND whether they are one- or two-sided<br>*Only common tests should be described solely by name; describe more complex techniques in the Methods section.*
☒	☐	A description of all covariates tested
☒	☐	A description of any assumptions or corrections, such as tests of normality and adjustment for multiple comparisons
☐	☒	A full description of the statistical parameters including central tendency (e.g. means) or other basic estimates (e.g. regression coefficient) AND variation (e.g. standard deviation) or associated estimates of uncertainty (e.g. confidence intervals)
☐	☒	For null hypothesis testing, the test statistic (e.g. *F*, *t*, *r*) with confidence intervals, effect sizes, degrees of freedom and *P* value noted<br>*Give P values as exact values whenever suitable.*
☒	☐	For Bayesian analysis, information on the choice of priors and Markov chain Monte Carlo settings
☒	☐	For hierarchical and complex designs, identification of the appropriate level for tests and full reporting of outcomes
☐	☒	Estimates of effect sizes (e.g. Cohen's *d*, Pearson's *r*), indicating how they were calculated

Our web collection on statistics for biologists contains articles on many of the points above.

Software and code

Policy information about availability of computer code

Data collection	No custom code was generated in this study.
Data analysis	For RNA-seq: Sequenced reads with low quality or adaptor contamination were removed. Clear reads were mapped to the human reference genome (hg19) using Hisat2 v2.2.1 software. To count the reads numbers mapped to each gene, featureCounts v2.0.6 was used. The fragments per kilobase of transcript per million mapped reads (FPKM) method was used to estimate expression levels. Differential expression analysis was performed using the DESeq2 R package (1.42.0).<br>For CUT&RUN ChIP-seq: The quality of ChIP-seq reads was checked using FastQC software (v0.11.9), and Trim Galore (v0.6.7-1) was used to remove adaptor sequences. After trimming, reads with a score >28 were aligned to the human hg19 reference genome and E. coli K12 MG1655 reference genome using Bowtie2 (v2.4.5). Sequencing data was normalized using a normalization factor calculated by sequencing the depth of E. coli Spike-in DNA reads. SAM files from reads aligned to hg19 were converted to BAM files, and PCR duplicates were removed using SAMtools (v1.12). Spike-in normalized bigwig and bedgraph files were generated by deepTools (v3.5.6). Bedgraph files were used for peak calling with MACS2 bdgpeakcall (v2.2.7.1). Highly reproducible peaks were identified using IDR (v2.0.4.2), and peaks overlapping blacklisted regions were removed using BEDTools (v2.30.0).<br>For ATAC-seq: Quality of ATAC-seq reads was checked using FastQC software (v0.11.9), and Trim Galore (v0.6.7-1) was used to remove adaptor sequences. After trimming, reads were aligned to the human hg19 reference genome using Bowtie2 (v2.4.5)–very-sensitive option. SAM files were converted to BAM files, and mitochondrial reads and PCR duplicates were removed using SAMtools (v1.12). BAM files were used for peak calling with MACS2 callpeak (v2.2.7.1). Highly reproducible peaks were identified using IDR (v2.0.4.2), and peaks overlapping blacklisted regions were removed using BEDTools (v2.30.0).

For manuscripts utilizing custom algorithms or software that are central to the research but not yet described in published literature, software must be made available to editors and reviewers. We strongly encourage code deposition in a community repository (e.g. GitHub). See the Nature Portfolio guidelines for submitting code & software for further information.

Data

Policy information about availability of data

All manuscripts must include a data availability statement. This statement should provide the following information, where applicable:

- Accession codes, unique identifiers, or web links for publicly available datasets
- A description of any restrictions on data availability
- For clinical datasets or third party data, please ensure that the statement adheres to our policy

The RNA-seq, CUT&RUN ChIP-seq, and ATAC-seq data generated in this study have been deposited in the GEO database under accession code GSE287918, https://www.ncbi.nlm.nih.gov/geo/query/acc.cgi?acc=GSE287918. All data are publicly available with no restrictions. The raw sequencing data are available in GEO, and processed data are also included.
In addition, publicly available datasets analyzed in this study are as follows: Raw HiChIP sequencing data for H3K27ac in Rv1 cells (GSE200168, https://www.ncbi.nlm.nih.gov/geo/query/acc.cgi?acc=GSE200168) and normalized AR ChIP-seq bigwig files from primary PCa and CRPC-PDX specimens (GSE130408, https://www.ncbi.nlm.nih.gov/geo/query/acc.cgi?acc=GSE130408). These datasets were reanalyzed to generate figures and results.
Source Data are provided with this paper.

Research involving human participants, their data, or biological material

Policy information about studies with human participants or human data. See also policy information about sex, gender (identity/presentation), and sexual orientation and race, ethnicity and racism.

Reporting on sex and gender	N/A
Reporting on race, ethnicity, or other socially relevant groupings	N/A
Population characteristics	N/A
Recruitment	N/A
Ethics oversight	N/A

Note that full information on the approval of the study protocol must also be provided in the manuscript.

Field-specific reporting

Please select the one below that is the best fit for your research. If you are not sure, read the appropriate sections before making your selection.

☒ Life sciences ☐ Behavioural & social sciences ☐ Ecological, evolutionary & environmental sciences

For a reference copy of the document with all sections, see nature.com/documents/nr-reporting-summary-flat.pdf

Life sciences study design

All studies must disclose on these points even when the disclosure is negative.

Sample size	Sample sizes were determined based on established guidelines and prior similar studies to ensure adequate statistical power. All sample sizes are clearly reported in the manuscript.
Data exclusions	No data was excluded from the analysis.
Replication	RNA-seq was performed using biological triplicates. ChIP-seq and ATAC-seq were performed using biological duplicates. RIME was performed with a single replicate and is considered exploratory rather than quantitative. qRT-PCR, ChIP-qPCR, and colony formation assays were performed in biological triplicate and independently repeated at least three times. Biological triplicates from one representative experiment were used for quantification and statistical analysis. Western blotting and co-immunoprecipitation experiments were validated using at least three independent samples.
Randomization	This study involved only in vitro experiments, and randomization was not applicable.
Blinding	Investigators were not blinded for any of the in vitro experiments. However, each experiment was independently repeated multiple times to ensure reproducibility.

Reporting for specific materials, systems and methods

We require information from authors about some types of materials, experimental systems and methods used in many studies. Here, indicate whether each material, system or method listed is relevant to your study. If you are not sure if a list item applies to your research, read the appropriate section before selecting a response.

Materials & experimental systems

n/a	Involved in the study	
☐	☒	Antibodies
☐	☒	Eukaryotic cell lines
☒	☐	Palaeontology and archaeology
☒	☐	Animals and other organisms
☐	☒	Clinical data
☒	☐	Dual use research of concern
☒	☐	Plants

Methods

n/a	Involved in the study	
☐	☒	ChIP-seq
☒	☐	Flow cytometry
☒	☐	MRI-based neuroimaging

Antibodies

Antibodies used

Rabbit polyclonal anti-BPTF (EMD Millipore, ABE24, Western blot 1:1000)
Rabbit polyclonal anti-BPTF (EMD Millipore, ABE1966, CUT&RUN 0.5 μg, ChIP-qPCR 3 μg, IP 3 μg)
Mouse monoclonal anti-BPTF (EMD Millipore, MABE443, IHC 1:300)
Rabbit polyclonal anti-AR (EMD Millipore, 06-680, Western blot 1:1000, CUT&RUN 0.5 μg, ChIP-qPCR 3 μg, IP 3 μg, RIME 10 μg)
Rabbit polyclonal anti-FOXA1 (EpiCypher, 13-2001, CUT&RUN 0.5 μg, ChIP-qPCR 3 μg, Western blot 1:1000)
Rabbit polyclonal anti-c-Myc (Cell Signaling 9402, Western blot 1:1000)
Mouse monoclonal anti-c-Myc (Santa Cruz, sc-40, Western blot 1:200)
Rabbit polyclonal anti-SMARCA1 (EpiCypher, 13-2005, CUT&RUN 0.5 μg)
Rabbit monoclonal anti-H3K27ac (EpiCypher, 13-0059, CUT&RUN 0.5 μg)
Rabbit mixed monoclonal anti-H3K4me1 (EpiCypher, 13-0040, CUT&RUN 0.5 μg)
Rabbit polyclonal anti-Flag (Sigma-Aldrich, F7425, Western blot 1:1000)
Mouse monoclonal anti-Flag (Sigma-Aldrich, F3165, Western blot 1:1000)
Rabbit monoclonal anti-HA (Cell Signaling, 3724, Western blot 1:1000)
Mouse monoclonal anti-Actin (Sigma-Aldrich, A5441, Western blot 1:10000)
Mouse monoclonal anti-Cas9 (Active Motif, 61957, ChIP-qPCR 3 μg)
Mouse monoclonal anti-RNA Pol Ⅱ (Active Motif, 39497, ChIP-qPCR 3 μg)
Rabbit IgG (EpiCypher, 13-0042, CUT&RUN 0.5 μg, ChIP-qPCR 3 μg)
Mouse IgG (Invitrogen, 02-6300, ChIP-qPCR 3 μg)
Goat anti-Rabbit IgG (H+L)-Alexa Fluor™ 680 (Invitrogen, A-21109, Western blot 1:1000)
Goat anti-Mouse IgG (H+L)-Alexa Fluor™ 680 (Invitrogen, A-21058, Western blot 1:1000)
Goat anti-Mouse IgG (H+L)-Alexa Fluor™ 790 (Invitrogen, A-11375, Western blot 1:1000)
Fluorescent TrueBlot anti-Rabbit IgG DyLight™ 680 (Rockland Immunochemicals, 18-4416-32, Western blot 1:1000)
Fluorescent TrueBlot Anti-Mouse IgG DyLight™ 680 (Rockland Immunochemicals, 18-4417-32, Western blot 1:1000)

Validation

Pre-validated antibodies were purchased from reputable commercial sources. We provided the Catalog number in the Supplementary Table3. The validation information on their webpages are listed below.

Rabbit polyclonal anti-BPTF, https://www.sigmaaldrich.com/US/en/product/mm/abe24
Rabbit polyclonal anti-BPTF, https://www.sigmaaldrich.com/US/en/product/mm/abe1966
Mouse monoclonal anti-BPTF, https://www.sigmaaldrich.com/US/en/product/mm/mabe443
Rabbit polyclonal anti-AR, https://www.sigmaaldrich.com/US/en/product/mm/06680
Rabbit polyclonal anti-FOXA1, https://www.epicypher.com/product/foxa1-hnf3a-cutana-cutrun-antibody/
Rabbit polyclonal anti-c-Myc, https://www.cellsignal.com/products/primary-antibodies/c-myc-antibody/9402?srsltid=AfmBOorQUwsYcHzLgi7uzNf8xG2ixifFQ6_UQnP7k87QpHTU1qvqgdl1
Mouse monoclonal anti-c-Myc, https://www.scbt.com/p/c-myc-antibody-9e10?srsltid=AfmBOoq29UL9FoYtvU83b9LRf1Jq2jY4HDkwlwJ29j1hcUe_t90nq8_V
Rabbit polyclonal anti-SMARCA1, https://www.epicypher.com/product/snf2l-smarca1-cutana-cutrun-antibody/
Rabbit monoclonal anti-H3K27ac, https://www.epicypher.com/product/h3k27ac-antibody-snap-certified-for-cutrun-and-cuttag/
Rabbit mixed monoclonal anti-H3K4me1, chrome-extension://efaidnbmnnnibpcajpcglclefindmkaj/https://www.epicypher.com/wp-content/uploads/2025/05/13-0040.pdf
Rabbit polyclonal anti-Flag, https://www.sigmaaldrich.com/US/en/product/sigma/f7425
Mouse monoclonal anti-Flag, https://www.sigmaaldrich.com/US/en/product/sigma/f3165
Rabbit monoclonal anti-HA, https://www.cellsignal.com/products/primary-antibodies/ha-tag-c29f4-rabbit-monoclonal-antibody/3724
Mouse monoclonal anti-Actin, https://www.sigmaaldrich.com/US/en/product/sigma/a5441
Mouse monoclonal anti-Cas9, https://www.activemotif.com/catalog/details/61757/cas9-antibody-mab-clone-8c1-f10
Mouse monoclonal anti-RNA Pol Ⅱ, https://www.activemotif.com/catalog/details/39097/rna-pol-ii-antibody-mab

Eukaryotic cell lines

Policy information about cell lines and Sex and Gender in Research

Cell line source(s)

Rv1 cells (also called CWR22Rv1 cells) were provided by Dr. James Jacobberger (Case Western Reserve University, Cleveland,

Cell line source(s)	Ohio). C4-2 cells were provided by Dr. Leland Chung (Cedars-Sinai Medical Center, Los Angeles, CA). LNCaP, VCaP, and SW620 cells were purchased from the American Type Culture Collection (ATCC).
Authentication	Cell lines were authenticated using short tandem repeat (STR) profiling.
Mycoplasma contamination	All cell lines were tested negative for mycoplasma by using PCR methods.
Commonly misidentified lines (See ICLAC register)	No commonly misidentified lines were used in this paper.

Clinical data

Policy information about clinical studies

All manuscripts should comply with the ICMJE guidelines for publication of clinical research and a completed CONSORT checklist must be included with all submissions.

Clinical trial registration	N/A
Study protocol	N/A
Data collection	N/A
Outcomes	N/A

Plants

Seed stocks	N/A
Novel plant genotypes	N/A
Authentication	N/A

ChIP-seq

Data deposition

☒ Confirm that both raw and final processed data have been deposited in a public database such as GEO.

☒ Confirm that you have deposited or provided access to graph files (e.g. BED files) for the called peaks.

Data access links *May remain private before publication.*	https://www.ncbi.nlm.nih.gov/geo/query/acc.cgi?acc=GSE287918
Files in database submission	Series GSE287901 (ATAC-seq)<br>GSM8754188 Rv1, control, rep1<br>GSM8754189 Rv1, control, rep2<br>GSM8754190 Rv1, BPTFKD, rep1<br>GSM8754191 Rv1, BPTFKD, rep2<br>GSM8754192 Rv1, SMARCA1KD, rep1<br>GSM8754193 Rv1, SMARCA1KD, rep2<br><br>Series GSE287902 (CUT&RUN ChIP-seq)<br>GSM8754194 Rv1, control, BPTF, rep1<br>GSM8754195 Rv1, control, BPTF, rep2<br>GSM8754196 Rv1, BPTFKD, BPTF, rep1<br>GSM8754197 Rv1, BPTFKD, BPTF, rep2<br>GSM8754198 Rv1, control, AR, rep1<br>GSM8754199 Rv1, control, AR, rep2<br>GSM8754200 Rv1, BPTFKD, AR, rep1<br>GSM8754201 Rv1, BPTFKD, AR, rep2<br>GSM8754202 Rv1, control, FOXA1, rep1<br>GSM8754203 Rv1, control, FOXA1, rep2<br>GSM8754204 Rv1, BPTFKD, FOXA1, rep1<br>GSM8754205 Rv1, BPTFKD, FOXA1, rep2<br>GSM8754206 Rv1, control, IgG<br>GSM8754207 Rv1, control, BPTF, forARKD, rep1

GSM8754208 Rv1, control, BPTF, forARKD, rep2
GSM8754209 Rv1, ARKD, BPTF, rep1
GSM8754210 Rv1, ARKD, BPTF, rep2
GSM8754211 Rv1, control, AR, forARKD, rep1
GSM8754212 Rv1, control, AR, forARKD, rep2
GSM8754213 Rv1, ARKD, AR, rep1
GSM8754214 Rv1, ARKD, AR, rep2
GSM8754215 Rv1, control, IgG, forARKD, forFOXA1KD
GSM8754216 Rv1, control, FOXA1, forFOXA1KD, rep1
GSM8754217 Rv1, control, FOXA1, forFOXA1KD, rep2
GSM8754218 Rv1, FOXA1KD, FOXA1, rep1
GSM8754219 Rv1, FOXA1KD, FOXA1, rep2
GSM8754220 Rv1, control, BPTF, forFOXA1KD, rep1
GSM8754221 Rv1, control, BPTF, forFOXA1KD, rep2
GSM8754222 Rv1, FOXA1KD, BPTF, rep1
GSM8754223 Rv1, FOXA1KD, BPTF, rep2
GSM8754224 Rv1, control, AR, forFOXA1KD, rep1
GSM8754225 Rv1, control, AR, forFOXA1KD, rep2
GSM8754226 Rv1, FOXA1KD, AR, rep1
GSM8754227 Rv1, FOXA1KD, AR, rep2
GSM8754228 Rv1, control, H3K27ac, rep1
GSM8754229 Rv1, control, H3K27ac, rep2
GSM8754230 Rv1, control, H3K4me1, rep1
GSM8754231 Rv1, control, H3K4me1, rep2
GSM9064092 Rv1, control, SMARCA1, rep1
GSM9064093 Rv1, control, SMARCA1, rep2

Series GSE287905 (RNA-seq)
GSM8754241 RNA-Seq, Rv1, control, rep1
GSM8754242 RNA-Seq, Rv1, control, rep2
GSM8754243 RNA-Seq, Rv1, control, rep3
GSM8754244 RNA-Seq, Rv1, BPTFKD, rep1
GSM8754245 RNA-Seq, Rv1, BPTFKD, rep2
GSM8754246 RNA-Seq, Rv1, BPTFKD, rep3
GSM8754247 RNA-Seq, Rv1, control, forSMARCA1.5KD, rep1
GSM8754248 RNA-Seq, Rv1, control, forSMARCA1.5KD, rep2
GSM8754249 RNA-Seq, Rv1, control, forSMARCA1.5KD, rep3
GSM8754250 RNA-Seq, Rv1, SMARCA1KD, rep1
GSM8754251 RNA-Seq, Rv1, SMARCA1KD, rep2
GSM8754252 RNA-Seq, Rv1, SMARCA1KD, rep3
GSM8754253 RNA-Seq, Rv1, SMARCA5KD, rep1
GSM8754254 RNA-Seq, Rv1, SMARCA5KD, rep2
GSM8754255 RNA-Seq, Rv1, SMARCA5KD, rep3
GSM8754256 RNA-Seq, Rv1, control, forFOXA1KD, rep1
GSM8754257 RNA-Seq, Rv1, control, forFOXA1KD, rep2
GSM8754258 RNA-Seq, Rv1, control, forFOXA1KD, rep3
GSM8754259 RNA-Seq, Rv1, FOXA1KD, rep1
GSM8754260 RNA-Seq, Rv1, FOXA1KD, rep2
GSM8754261 RNA-Seq, Rv1, FOXA1KD, rep3

Genome browser session (e.g. UCSC)

We only deposit data on GEO and not in the Genome Browser session.

Methodology

Replicates

Two biological replicates (Rv1 cells) were used

Sequencing depth

All samples were sequenced to a depth of 20 million reads per library in paired-end mode (150 bp).

Antibodies

BPTF, EMD Millipore ABE1966
AR, EMD Millipore 06-680
FOXA1, EpiCypher 13-2001
H3K27ac, EpiCypher 13-0059
H3K4me1, EpiCypher 13-0040
SMARCA1, EpiCypher 13-2005
Rabbit IgG, EpiCypher 13-0042

Peak calling parameters

For CUT&RUN ChIP-seq, peak calling was performed using MACS2 (macs2 bdgpeakcall -i [input file] -c [cutoff] (7 for BPTF, 15 for AR, 8 for FOXA1, 2 for SMARCA1) -l 200 -g 150
For ATAC-seq, peak calling was performed using MACS2 (macs2 callpeak -f BAMPE -g hs -B --keep-dup all --cutoff-analysis -t [input file])

Data quality

FastQC

Software

For RNA-seq: Hisat2 (v2.2.1), featureCounts (v2.0.6), DESeq2 R package (1.42.0).

Software

For CUT&RUN ChIP-seq and ATAC-seq: FastQC (v0.11.9), Trim Galore (v0.6.7-1), Bowtie2 (v2.4.5), SAMtools (v1.12), deepTools (v3.5.6), MACS2 (v2.2.7.1), IDR (v2.0.4.2), BEDTools (v2.30.0).
